# A Mycophenolic Acid Concentration-Time Curve (0-12 Hours) to Maintain Remission Without Recurrent Infection in C1q Nephropathy: A Case Report

**DOI:** 10.7759/cureus.87544

**Published:** 2025-07-08

**Authors:** Marika Ishii, Takahiro Kanai, Jun Aoyagi, Tomomi Maru, Toshihiro Tajima

**Affiliations:** 1 Pediatrics, Jichi Medical University, Shimotsuke, JPN

**Keywords:** bk virus, c1q nephropathy, mycophenolate mofetil, mycophenolic acid, recurrent infection, steroid-resistant nephrotic syndrome, therapeutic drug monitoring

## Abstract

C1q nephropathy (C1qN) presenting with steroid-resistant nephrotic syndrome (SRNS) often demands immunosuppressants to maintain remission. Mycophenolate mofetil (MMF), a prodrug of mycophenolic acid (MPA), is one of the immunosuppressants used. However, MMF increases susceptibility to infection, which can lead to severe complications in some cases. Therefore, the optimal blood concentration of MMF for C1qN remains undefined. A three-year-old boy presented with SRNS due to C1qN and was treated with prednisolone (PSL) and MMF at 720 mg/day, achieving remission. However, the patient developed BK virus viruria and experienced a relapse of nephrotic syndrome (NS) simultaneously. After the MMF dose was reduced to 600 mg/day, the patient entered sustained remission without recurrent infections. Ultimately, he was able to maintain remission with only 600 mg/day of MMF, without the need for steroids. During the second remission, the area under the MPA concentration-time curve from 0 to 12 hours (MPA-AUC_0-12_) was measured at 53.2 μg·h/mL, suggesting that this level may be sufficient to maintain remission while minimizing the risk of infection, and could serve as a reference point for determining the optimal blood concentration in C1qN treatment. To our knowledge, this is the first report to suggest a potentially optimal MPA-AUC_0-12_ for maintaining remission in C1qN without recurrent infection. Further studies of MPA-AUC_0-12_ for C1qN are warranted to confirm this result.

## Introduction

C1q nephropathy (C1qN) is a glomerular disease entity with a reported frequency ranging from 0.2% to 16% among patients undergoing renal biopsy and is more frequently observed in children [1]. The pathological findings are defined by dominant mesangial C1q deposition on immunofluorescence, with equal or lesser intensity of complement 3 (C3), immunoglobulin M (IgM), and immunoglobulin G (IgG) deposition. Light microscopic findings are diverse, such as minor glomerular abnormalities, focal segmental glomerulosclerosis, or mesangial proliferative glomerulonephritis [1,2].

C1qN often presents as steroid-resistant nephrotic syndrome (SRNS) and frequently requires immunosuppressive agents to maintain remission. Mycophenolate mofetil (MMF) is one of the immunosuppressants used for this purpose [3]. However, MMF increases susceptibility to both de novo and recurrent infections [4]. For example, activation of the BK virus is often observed in patients receiving MMF therapy and has been reported to cause renal dysfunction in kidney transplant recipients [5,6]. Therefore, the MMF dosage must achieve an optimal blood concentration that maintains remission in C1qN while minimizing the risk of infection. Nonetheless, the optimal blood concentration of MMF for this purpose remains unclear.

MMF is an ester prodrug of mycophenolic acid (MPA), an immunosuppressant [7]. The effects of MPA depend on its blood concentration [8]. Therefore, the optimal MMF dose should be analyzed in terms of the blood MPA concentration. In analyzing the blood MPA concentration, the enterohepatic circulation of MPA should be considered, because it leads to a second peak occurring between four and eight hours after MMF administration [9]. Therefore, the blood MPA concentration should be analyzed by the area under the concentration-time curve from 0 to 12 hours (AUC_0-12_) rather than by single trough concentrations. In fact, the incidence of acute rejection in kidney transplantation was significantly associated with MPA-AUC_0-12_ (p < 0.02), whereas no significant association was found between rejection and MPA trough levels (p = 0.21) [10].

Here, we report the remission-phase MPA-AUC_0-12_ in a case of C1qN that was maintained in remission on MMF without recurrent reactivation of BK virus.

## Case presentation

**Figure 1:**
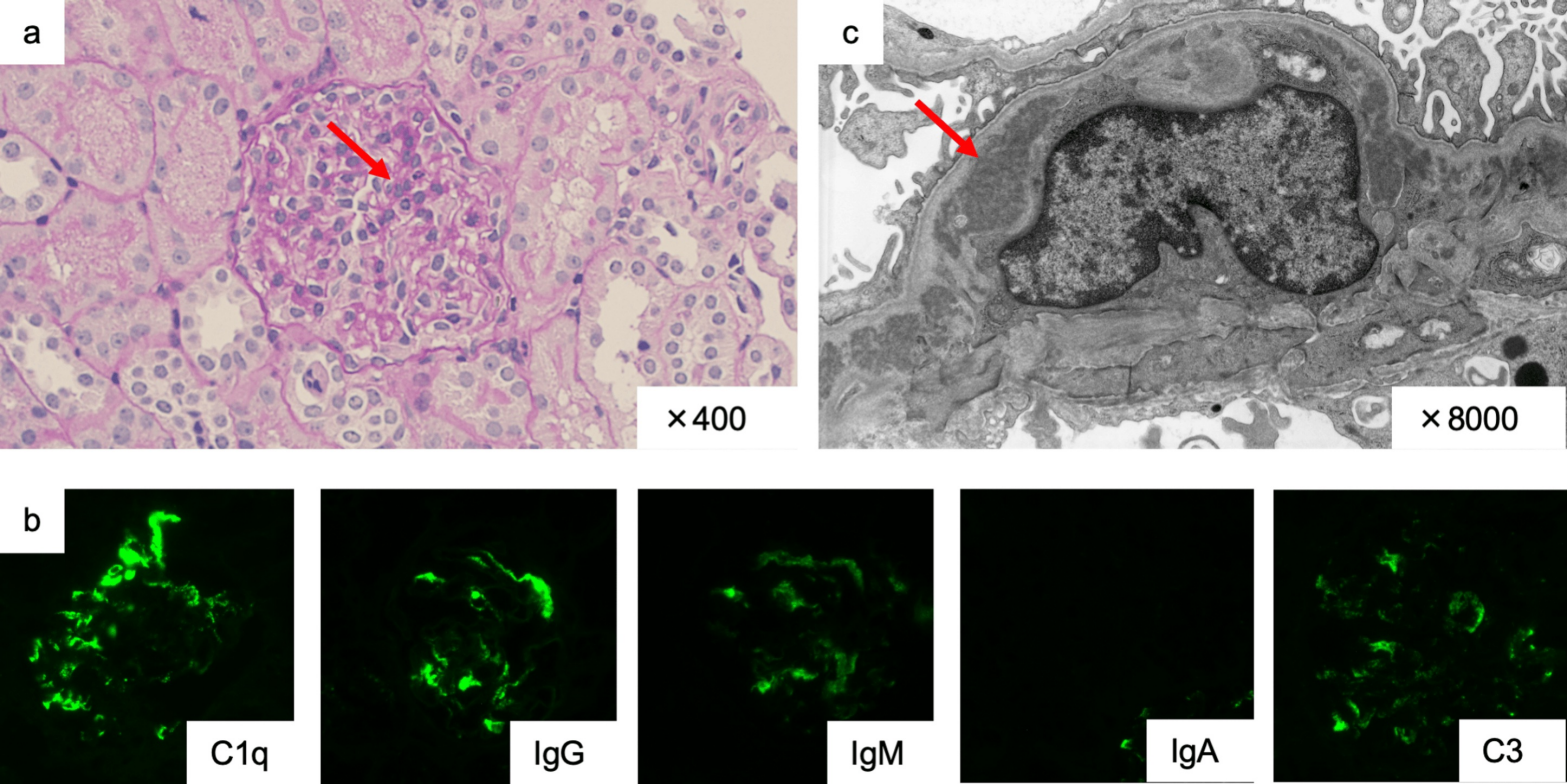
Renal biopsy findings a) Periodic acid–Schiff staining shows mild diffuse mesangial proliferation (arrow). b) Immunofluorescence reveals dominant mesangial staining for C1q compared to other immunoglobulins. c) Electron microscopy shows electron-dense deposits in the mesangial area (arrow). IgG, immunoglobulin G; IgM, immunoglobulin M; IgA, immunoglobulin A; C3, complement 3

A three-year-old boy with idiopathic SRNS [11] was referred to our hospital. There was no relevant medical history. Physical examination and laboratory findings were as follows: height, 92.5 cm (+0.3 standard deviation for a standard Japanese boy); weight, 15.4 kg (+1.0 standard deviation for a standard Japanese boy), with generalized edema; blood pressure, 105/63 mmHg; serum creatinine (s-Cr), 0.34 mg/dL (2.5th to 97.5th percentile range for Japanese three-year-old children: 0.21-0.37 mg/dL); serum albumin (s-Alb), 0.7 g/dL (reference range: 4.1-5.1 g/dL); total cholesterol, 416 mg/dL (reference range: 142-248 mg/dL); urine protein, 2,845 mg/dL (reference range: <5 mg/dL); urine protein-to-creatinine ratio (UP/UCr), 27.86 g/g×Cr (reference value: <0.15 g/g×Cr); and selectivity index, 0.28 (values >0.2 indicate non-selective proteinuria). Hematuria was not detected. Treatment was initiated according to the Kidney Disease: Improving Global Outcomes (KDIGO) guidelines [11] and International Study of Kidney Disease in Children (ISKDC) [12]; however, prednisolone (PSL) 28 mg/day (2 mg/kg/day) failed to induce remission at four weeks after initiation, and the patient was diagnosed with SRNS. His renal biopsy revealed the following findings: on light microscopy, mesangial cell proliferation in five of the 108 glomeruli observed (Figure 1a); on immunofluorescence microscopy, dominant mesangial staining for C1q (Figure 1b), with less intense staining for IgG, C3, IgM, and immunoglobulin A (IgA) compared to C1q; and on electron microscopy, electron-dense deposits in the mesangium (Figure 1c).

There were no clinical symptoms suggestive of systemic lupus erythematosus. These pathological and clinical findings were compatible with the C1qN diagnostic criteria proposed by Jennette and Hipp [2]. The pathogenesis of C1qN involves the deposition of C1q, IgG, IgM, and C3 in the mesangial cells [2,13]. Therefore, to suppress IgG and/or IgM production and then inactivate the classical pathway through C1q, MMF at a dosage of 720 mg/day (1176 mg/m^2^/day) was initiated with PSL 28 mg/day. The MMF dose was determined with reference to the 1200 mg/m²/day recommended by KDIGO for nephrotic syndrome (NS) [11]. In accordance with the guidelines, the PSL dose was tapered while being co-administered with MMF [11]. This led to remission on the 15th day after MMF initiation. However, on the 33rd day after MMF initiation (the 19th day of remission), decoy cells were detected in his urine for the first time in this clinical course, and the qualitative test for BK virus deoxyribonucleic acid (DNA) in his urine was positive. Testing for the JC virus in the urine was also performed but it was negative. A renal biopsy was not performed for this relapse, and, therefore, SV40 staining was not performed on renal tissue. Additionally, the NS relapsed, with UP/UCr of 8.3 g/g×Cr. At this time, the patient was receiving the same dosage of MMF (720 mg/day) but a reduced dose of PSL at 18 mg/day (1.3 mg/kg/day). Therefore, the MMF dosage was reduced from 720 mg/day to 600 mg/day (980 mg/m²/day), and the PSL dosage was increased from 18 mg/day to 28 mg/day. This combination therapy with MMF and PSL induced remission again on the eighth day after the relapse of NS. At this point, the BK virus load in the urine reached 4.9×10³ copies/mL. After 284 days on the reduced MMF dose, the viral load decreased to 1.4×10² copies/mL and became undetectable after 457 days. The patient has had no recurrent infections since then. Following the reduction of MMF to 600 mg/day, the BK virus load showed a continuous downward trend, and no adverse events, including NS relapse, were observed. Therefore, the MMF dosage of 600 mg/day was maintained. While MMF was continued at this dosage, PSL was gradually tapered and ultimately discontinued 19 months after achieving re-remission. The patient has remained relapse-free for 31 months since achieving remission.

**Figure 2:**
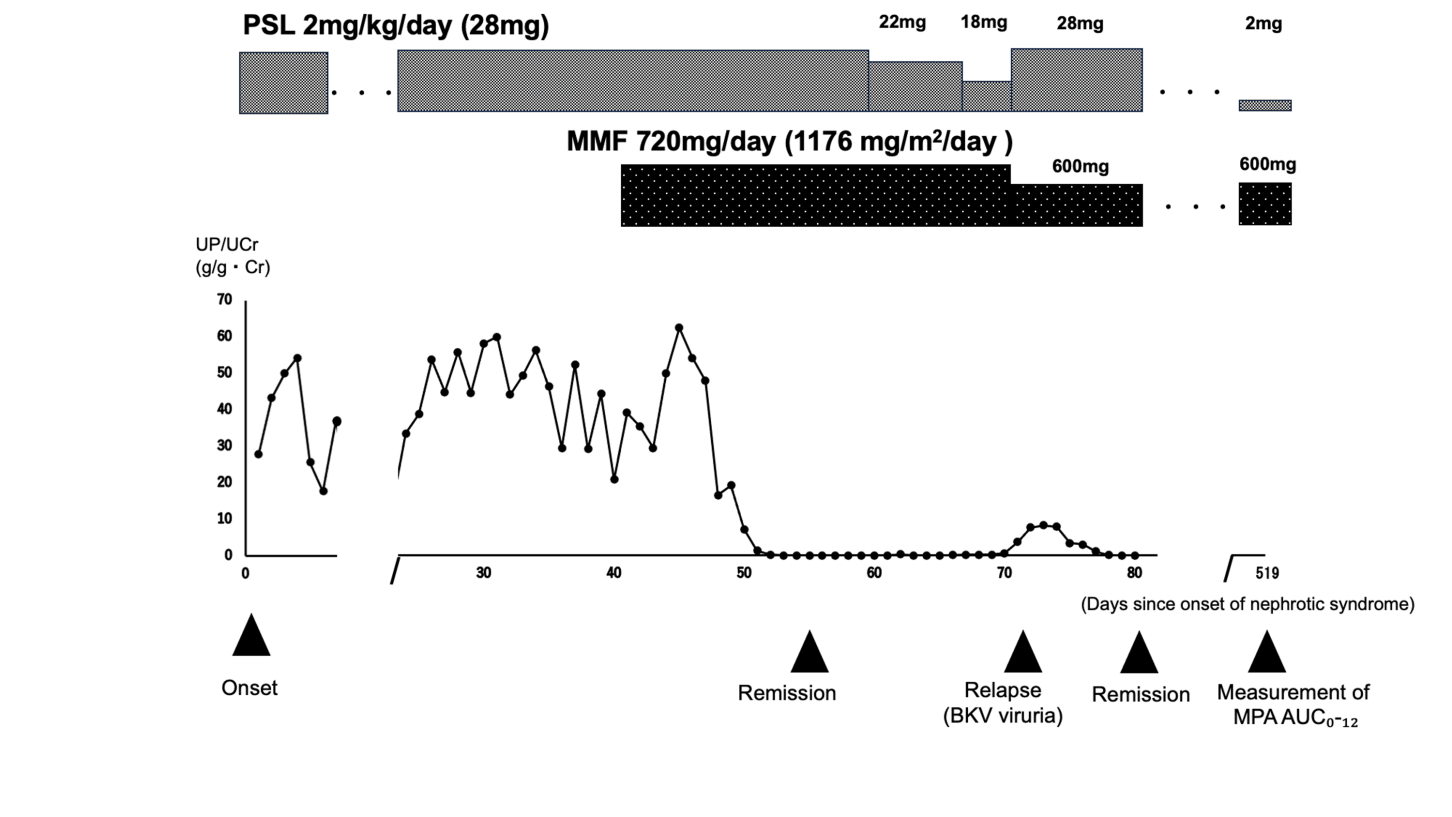
Clinical course PSL, prednisolone; MMF, mycophenolate mofetil; UP/UCr, urine protein:creatinine ratio; BKV, BK virus; AUC_0-12_, area under the concentration-time curve from 0 to 12 hours; MPA, mycophenolic acid

While the second remission was maintained, the MPA-AUC₀-₁₂ was measured at 449 days after MMF dose reduction. Blood samples were collected at zero, one, three, and six hours after MMF administration, and MPA levels were determined using liquid chromatography-tandem mass spectrometry (LC-MS/MS). The analysis was performed using a Nihon Waters Xevo TQ-S micro system with a Nihon Waters XBridge Premier BEH C18 column (2.5 μm, 2.1×50 mm). The MPA concentrations at each time point were as follows: 1.8 μg/mL at trough, 23.3 μg/mL at one hour post-dose, 1.5 μg/mL at three hours post-dose, and 4.0 μg/mL at six hours post-dose (Table 1). The estimated MPA-AUC₀-₁₂, calculated using the equation proposed by Sobiak, was 53.2 μg·h/mL [14]. This equation was developed and validated based on full 0-12 hour pharmacokinetic profiles collected from pediatric patients with NS [14]. The overall clinical course of this case is illustrated in Figure 2.

**Table 1:** Plasma MPA concentrations at recurrence The equation for the estimated MPA-AUC_0-12_ proposed by Sobiak et al. is the following: MPA-AUC_0-12 _= 7.10+1.21×C1+3.75×C3+3.08×C6 [14]. AUC_0-12_, area under the concentration-time curve from 0 to 12 hours; MPA, mycophenolic acid

Plasma MPA concentration at recurrence				
Trough (μg/mL)	After one hour (μg/mL)	After three hours (μg/mL)	After six hours (μg/mL)	AUC_0-12_ (μg×h/mL)
1.8	23.3	1.5	4.0	53.2

## Discussion

The C1qN case presented in this report was maintained in remission on MPA-AUC_0-12_ of 53.2 μg·h/mL, without recurrent reactivation of BK virus. To our knowledge, this is the first report to suggest a potential target range for MPA-AUC_0-12_ in C1qN to maintain remission without recurrent reactivation of BK virus. 

There are no treatment guidelines for C1qN. In C1qN, C1q binds with IgG and/or IgM, which are subsequently deposited in mesangial cells [2,13]. Following this, the complex of C1q and IgG and/or IgM activates the classical complement pathway [13,15], which is believed to lead to mesangial cell proliferation. MMF can suppress the production of IgG and IgM. This pharmacological effect could block or reduce the mesangial cell proliferation associated with activation of the classical pathway, thereby inducing and maintaining C1qN remission. Thus, MMF may have greater potential to induce and maintain remission in C1qN compared with cyclosporine. However, the optimal blood concentration of the active form of MMF, MPA, especially the MPA-AUC_0-12_, is still unclear. 

Currently, the most commonly cited target ranges for MPA-AUC_0-12_ come from the field of solid organ transplantation, where levels of 30-60 μg·h/mL are considered effective for preventing rejection without increasing adverse events [8]. For other conditions, no clear standards have been established, and to date, no reports have described an optimal MPA-AUC_0-12_ for C1qN. While not a comparative study, Gellermann et al. reported that an MPA-AUC_0-12_ of 60-80 μg·h/mL was required to maintain remission in cases of SRNS [16]. In our case, the MPA-AUC_0-12_ of 53.2 μg·h/mL maintained remission of C1qN without recurrent reactivation of BK virus. This suggests that the optimal MPA-AUC_0-12_ for maintaining remission may differ between C1qN and other forms of SRNS, with a lower requirement in C1qN. Additionally, the MPA-AUC_0-12_ of 53.2 μg·h/mL was associated with the disappearance of BK virus in the urine. Moreover, in our case, 720 mg/day of MMF resulted in BK virus viruria, which could be associated with the recurrence of NS. This suggests that physicians should recognize that high blood MPA concentrations can lead to reactivation of viral infections. In fact, Borrows et al. reported that a trough MPA level of 3.20 mg/L or higher was associated with an increased risk of viral infections, including BK virus [4]. In our case, the trough level was 1.8 mg/L (1.8 μg/mL) at an MMF dose of 600 mg/day. 

The infection may have been precipitated by monitoring the patient during the four-week period of PSL therapy. However, it is rare for an infection to be triggered during a four-week observation period under PSL treatment. In fact, treatment with PSL at 2 mg/kg/day for four weeks is a standard regimen in accordance with guidelines such as KDIGO [11] and ISKDC [12]. On the other hand, excessive use of MMF can cause BK virus infection [4]. In this case, BK virus infection improved even after the steroid dose was increased and the MMF dose was reduced, suggesting that MMF had a greater impact than PSL on the reactivation of BK virus. However, it has been reported that the risk of BK virus reactivation increases when steroids are used in combination with MMF [17], and it is therefore possible that the combination of MMF and steroids contributed to BK virus reactivation. If the optimal therapeutic range of MPA blood concentrations can be more clearly defined in the future, the use of MMF may allow for safer and more effective tapering of PSL, potentially reducing the risk of steroid-related adverse effects.

Factors that affect blood MPA concentrations include co-administered drugs (e.g., steroids and cyclosporine), renal function, dietary intake, and formulation differences (e.g., MMF vs. enteric-coated mycophenolate sodium) [8], all of which contribute to inter-individual variability and underscore the importance of monitoring MPA concentrations in clinical practice. Furthermore, it is known that young children require relatively higher doses of MMF per body weight to achieve blood MPA concentrations comparable to those in older children [18]. This difference is associated with changes in MPA clearance [18]. Therefore, especially in younger children, it is essential to measure blood MPA concentrations and optimize MMF dosing accordingly. In addition, due to the influence of enterohepatic circulation, blood MPA levels exhibit a second peak four to eight hours after MMF administration [9]. It has been reported that there is no consistent correlation between MPA-AUC_0-12_ and the MPA trough level [8], and in the field of kidney transplantation, it has been considered preferable to assess MPA exposure using MPA-AUC_0-12_ rather than a single trough level [10]. In diseases such as NS, including C1qN, it is also possible that MPA-AUC_0-12_, rather than trough levels, may be preferable for preventing disease recurrence and viral reactivation. At present, we can only suggest that MPA-AUC_0-12_ may be a more reliable indicator than trough levels for the prevention of BK virus reactivation and recurrence of C1qN. Further accumulation of data in this area and individualized MMF dosing based on blood MPA concentrations are desirable.

This report is limited by being based on a single case, and therefore, the optimal blood MPA concentration for C1qN cannot be determined from this case alone. However, in this case, although treatment was initiated at the commonly recommended pediatric MMF dose, excessive immunosuppression appeared to have caused reactivation of the BK virus, which triggered a relapse of NS. Remission was subsequently maintained with a lower MMF dose. This suggests that monitoring MPA concentrations may be clinically useful for simultaneously preventing both recurrent infections and relapse of NS. The MPA-AUC_0-12_ value obtained in this report may serve as a useful reference for future studies aimed at establishing optimal blood concentration ranges not only in post-transplant patients but also in diseases such as C1qN that present with NS. To validate this finding, further accumulation of MPA-AUC_0-12_ data from additional cases treated based on this value is warranted.

## Conclusions

In this single case, we identified the MPA-AUC_0-12_ associated with maintaining remission of C1qN without recurrent infection. This finding represents an initial step toward identifying the optimal MPA-AUC_0-12_ that minimizes infection risk during treatment of C1qN. In this case, adverse events occurred even at conventionally recommended doses of MMF for pediatric patients, and remission was maintained with a lower MMF dose than typically recommended. These observations suggest that monitoring MPA blood concentrations may be useful. As this report is based on a single case, further investigation of MPA-AUC_0-12_ is warranted to confirm the generalizability of these findings.
